# Antifungal efficacy of isavuconazole and liposomal amphotericin B in a rabbit model of *Exserohilum rostratum* meningoencephalitis: A preclinical paradigm for management of CNS phaeohyphomycosis

**DOI:** 10.1093/mmy/myaa102

**Published:** 2020-12-12

**Authors:** Vidmantas Petraitis, Ruta Petraitiene, Aspasia Katragkou, Bo Bo Win Maung, Patriss W Moradi, Gittel E Sussman-Straus, Ethan Naing, Laura L Kovanda, Malcolm A Finkelman, Thomas J Walsh

**Affiliations:** Transplantation-Oncology Infectious Diseases Program, Division of Infectious Diseases, Department of Medicine, Weill Cornell Medicine of Cornell University, New York, New York, USA; Transplantation-Oncology Infectious Diseases Program, Division of Infectious Diseases, Department of Medicine, Weill Cornell Medicine of Cornell University, New York, New York, USA; Transplantation-Oncology Infectious Diseases Program, Division of Infectious Diseases, Department of Medicine, Weill Cornell Medicine of Cornell University, New York, New York, USA; New York Presbyterian Brooklyn Methodist Hospital, Brooklyn, New York, USA; Transplantation-Oncology Infectious Diseases Program, Division of Infectious Diseases, Department of Medicine, Weill Cornell Medicine of Cornell University, New York, New York, USA; Transplantation-Oncology Infectious Diseases Program, Division of Infectious Diseases, Department of Medicine, Weill Cornell Medicine of Cornell University, New York, New York, USA; Transplantation-Oncology Infectious Diseases Program, Division of Infectious Diseases, Department of Medicine, Weill Cornell Medicine of Cornell University, New York, New York, USA; Transplantation-Oncology Infectious Diseases Program, Division of Infectious Diseases, Department of Medicine, Weill Cornell Medicine of Cornell University, New York, New York, USA; Astellas Pharma Global Development, Inc., Northbrook, Illinois, USA; Associates of Cape Cod, Inc., East Falmouth, Massachusetts, USA; Transplantation-Oncology Infectious Diseases Program, Division of Infectious Diseases, Department of Medicine, Weill Cornell Medicine of Cornell University, New York, New York, USA; Department of Microbiology & Immunology, Weill Cornell Medicine of Cornell University, New York, New York, USA; Department of Pediatrics, Weill Cornell Medicine of Cornell University, New York, New York, USA

**Keywords:** experimental CNS phaeohyphomycosis, isavuconazole, liposomal amphotericin B, rabbits

## Abstract

Treatment options for *Exserohilum rostratum* meningoencephalitis and other causes of phaeohyphomycosis of the central nervous system (CNS) are limited, while mortality and morbidity remain high. We therefore evaluated isavuconazole, a new antifungal triazole in comparison to liposomal amphotericin B (LAMB), in vitro and in the rabbit model of *Exserohilum rostratum* meningoencephalitis. We hypothesized that isavuconazole alone or in combination with LAMB or micafungin may be alternative options for treatment of CNS phaeohyphomycosis. We therefore investigated the in vitro antifungal activity of isavuconazole alone or in combination with amphotericin B deoxycholate (DAMB) or micafungin and efficacy of treatment with isavuconazole and LAMB in a rabbit model of experimental *E. rostratum* meningoencephalitis. Combination checkerboard plates were used to determine the minimum inhibitory concentrations, minimal lethal concentrations, fractional inhibitory concentration indices, and Bliss surface analysis of isavuconazole and amphotericin B deoxycholate (DAMB), either alone or in combination. As there were no in vitro synergistic or antagonistic interactions for either combination of antifungal agents against the *E. rostratum* isolates, in vivo studies were conducted with isavuconazole and LAMB as monotherapies. Rabbits were divided in following groups: treated with isavuconazole at 60 mg/kg/d (ISAV60), LAMB at 5.0 (LAMB5), 7.5 (LAMB7.5), and 10 mg/kg/d (LAMB10), and untreated controls (UC). In ISAV60-, LAMB5-, LAMB7.5-, and LAMB10-treated rabbits, significant reductions of fungal burden of *E. rostratum* in cerebral, cerebellar, and spinal cord tissues (*P *< 0.01) were demonstrated in comparison to those of UC. These antifungal effects correlated with significant reduction of CSF (1→3)-β-D-glucan levels vs UC (*P *< 0.05). These data establish new translational insights into treatment of CNS phaeohyphomycosis.

## Introduction

Mould infections of the central nervous system (MICNS) are life-threatening diseases with high mortality and morbidity rates.^1^ Among the therapeutic options available for treatment of MICNS, those for management of CNS aspergillosis have been well described,^2^^,^^3^ however, considerably less is known about the therapeutic options for CNS phaeohyphomycosis.^4^^,^^5^ Although voriconazole or liposomal amphotericin B have been recognized as standards of care in treatment of CNS phaeohyphomycosis, both agents have efficacy, safety, and tolerability limitations.

Revankar et al reported that among 26 patients with disseminated phaeohyphomycosis, including 12 with CNS infection, 16 received two or more antifungal agents. Despite the frequent use of combination antifungal therapy, patients with disseminated phaeohyphomycosis had a clinical response of 31% and mortality of 69%. An earlier study of CNS phaeohyphomycosis by Revankar and colleagues also found that overall mortality in this infection was 74%.^4^

Isavuconazole is a recently introduced antifungal triazole with proven clinical efficacy in the treatment of invasive infections caused by *Aspergillus* and Mucorales.^6^^,^^7^ Given the broad spectrum of antifungal activity against pathogenic moulds, we hypothesized that isavuconazole may be a viable alternative for treatment of CNS phaeohyphomycosis.^8^^,^^9^ Moreover, we considered that the combination of isavuconazole plus micafungin or the combination of isavuconazole plus liposomal amphotericin B (LAMB) might be synergistic for treatment of CNS phaeohyphomycosis.

*Exserohilum rostratum* was the etiological agent of the multi-state outbreak in the USA of CNS phaeohyphomycosis caused by the administration of a contaminated methylprednisolone injection preparation.^10^^-^^13^ As more than 600 cases of *Exserohilum rostratum* CNS infection were identified, this infection constitutes the most widely treated form of CNS phaeohyphomycosis documented thus far.^14^

We therefore studied the antifungal activity of isavuconazole alone and in combination with micafungin or amphotericin B deoxycholate (DAMB) against of *E. rostratum* in vitro and, based upon these findings, proceeded to investigate the efficacy of isavuconazole alone and LAMB alone for the treatment of CNS phaeohyphomycosis in an immunocompromised rabbit model of *E. rostratum* meningoencephalitis.

## Methods

### Organisms and *in vitro* studies

Five isolates of *E. rostratum* (12-2725, 12–2786, 12–2809, 12–2838, and 12–2840), obtained from patients suffering from *E. rostratum* meningoencephalitis, as the result of the corticosteroid injection related outbreak, were used in the in vitro experiments.^15^

The CLSI M38-A3 methodology was applied for inoculum preparation of each *E. rostratum* isolate.^16^ Mould isolates were subcultured onto a potato dextrose agar slant (PDA) and incubated at 37°C for 24 h, and then stored for additional 5-7 days at ambient temperature. The conidial suspensions of 0.4-5 × 10^4^ CFU/mL were prepared using a 0.025% Tween 20 solution in 0.9% normal saline. The conidial suspensions were measured with a spectrophotometer at 530-nm wavelength and brought to 80-82% transmittance (%T) readings.

We further studied the in vitro antifungal activity of isavuconazole in combination with micafungin or amphotericin B against of *E. rostratum* based on their activity when administered alone and from previously published data on combination efficacy against *Aspergillus fumigatus*.^17^^,^^18^

The combination checkerboard plates were prepared according to the CLSI recommendations. An aliquot of 100 μL of the vortexed suspension was inoculated into each well of a 96-well flat-bottom microplate containing 100 μL of single drugs and two-drug combinations of isavuconazole (ISAV; Astellas Pharma Global Development, Inc., Northbrook, IL) plus micafungin (MFG), or ISAV plus deoxycholate amphotericin B (DAMB) (X-GEN Pharmaceuticals, Big Flats, NY). The tested concentrations in checkerboard dilutions ranged from 0.0625 to 32 μg/mL for ISAV, from 0.5 to 32 μg/mL for MFG, and from 0.125 to 8 μg/mL for DAMB. After 48-h incubation at 37°C, optical density (OD) measurements were recorded at 530-nm wavelength. Bliss surface interaction and fractional inhibitory concentration indifference (FICI) indices were then calculated. The minimal inhibitory concentrations (MICs) and minimal lethal concentrations (MLCs) of antifungal drugs were determined, and fractional inhibitory concentration (FIC) indices were calculated.^19^ The MICs were determined as optically clear wells. The MLCs were determined as wells that produced no fungal growth once plated on PDA plates. Additional analysis was performed using Bliss surface interaction analysis, as previously described.^19^

### Inoculum preparation and inoculation procedures for in vivo studies

The previously studied *E. rostratum* isolates 12–2725 and 12–2809 were used in all in vivo experiments. In order to detect differences in the in vivo response, we selected two isolates based on our preliminary in vitro data: one isolate with lower MIC to isavuconazole and the other with higher MIC. Although the MIC's were different for two isolates, we did not observe significant differences in the in vivo response to treatment. Therefore, the data were pooled, presented, and analyzed in aggregate.

Inoculum preparation and inoculation were performed as previously described.^20^ In brief, *E. rostratum* isolates were subcultured onto PDA plates, incubated at 37°C for 24 h, and maintained inverted for 1-2 weeks. Then multiple plates were filled with 10 mL of 0.025% Tween-20 saline solution and gently scraped with a 10-mL pipette. The resulting suspensions of *E. rostratum* were combined into 50-mL conical tubes. After tubes were centrifuged, the supernatant and floating fungal elements were removed leaving 5 mL in the tubes, combined, and conidia were resuspended in 0.025% Tween-20 saline solution into a final volume, based upon the number of *E. rostratum* conidia counted with a hemacytometer. Following vigorous vortexing, aggregation of the conidia was negligible but when present, was considered as a single infecting unit. The final concentration of *E. rostratum* was 1.0 × 10^6^ CFU of conidia. 500 μL of inoculum was administered to anesthetized rabbits, as previously described,^20^ intracisternally in the amount of 500 μL for each rabbit, after equal amount of CSF (500 μL) was withdrawn from the cisterna magna before inoculation. The inoculum concentrations were verified by serial dilutions on Sabouraud glucose agar plates.

### Animals

Female New Zealand White rabbits (Envigo RMS, Inc. Denver, PA) were used for all studies. At the time of intracisternal inoculation, rabbits weighted 2.6-3.6 kg. All groups were included in the same experiment, which was conducted in replicate. All rabbits were monitored under humane care and use standards in facilities, accredited by the Association for Assessment and Accreditation of Laboratory Animal Care International, according to the guidelines of the National Research Council for the care and use of laboratory animals.^21^ The experiments were approved by the Animal Care and Use committee of the Weill Cornell Medicine, New York, NY. Rabbits had an indwelling Silastic central venous catheter, placed under general anesthesia, for the atraumatic collection of diagnostic samples and the parenteral administration of investigational agents, as well as compounds required to induce and support neutropenia.

### Immunosuppression and maintenance of neutropenia

Induction of profound persistent neutropenia and immunosuppression was initiated 6 days before intracisternal inoculation and maintained using cytarabine (Ara-C) (Cytarabine injection; Hospira, Inc., Lake Forest, IL) at a dosage of 440 mg/m^2^ for 5 consecutive days (days 1-5) with subsequent administration on days 8-9 and 13-14, as previously described.^20^ Methylprednisolone (Solu-Medrol^®^, Pfizer Inc., NY, NY) was administered at 5 mg/kg of body weight for immunosuppression from day 1 to the end of experiment. Ceftazidime (75 mg/kg i.v. twice daily; Glaxo Pharmaceuticals, Division of Glaxo Inc., Research Triangle Park, NC), gentamicin (5 mg/kg i.v. every other day; Elkins-Sinn, Inc., Cherry Hill, NJ), and vancomycin (15 mg/kg i.v. daily; Abbott Laboratories, North Chicago, IL) were administered intravenously beginning on day 4 of the study to prevent bacterial infections. Vancomycin (50 mg/L) was added to the drinking water to prevent antibiotic-associated diarrhea due to *Clostridium spiroforme* from the beginning of induction of neutropenia.

### Inoculum-response studies

In order to define the optimal *E. rostratum* inoculum, initial studies were conducted in three inoculum groups of four rabbits each to investigate the fungal burden in cerebral, cerebellar, and spinal cord tissues after intracisternal inoculation of defined suspensions of 1.0 × 10^2^, 1.0 × 10^4^, and 1.0 × 10^6^ conidia. A total of four animals were used in each of the three inoculum groups. Following euthanasia at the end of the study or because of humane end points, cerebral, cerebellar, and spinal cord tissue samples were harvested and quantitative fungal cultures were performed.

### Antifungal agents and experimental groups

Isavuconazole was administered as an oral formulation of isavuconazonium sulfate. Prodrug isavuconazonium sulfate powder was dissolved in sterile water and mixed with the same amount of 5% dextrose injection solution (D5W; Baxter Healthcare Corp., Deerfield, IL) for oral administration. The oral dosage of 60 mg/kg/d was based upon the active moiety isavuconazole. LAMB (AmBisome, Astellas Pharma Global Development, Inc., Northbrook, IL) was administered intravenously in dosages of 5, 7.5, and 10 mg/kg/d.

Antifungal therapy was initiated on day 7 (1 day after inoculation) and continued throughout the experiment for 13 days in surviving rabbits. Study groups consisted of isavuconazole at 60 mg/kg/d (ISAV60, *n* = 8), LAMB at 5.0 (LAMB5, *n* = 8), 7.5 (LAMB7.5, *n* = 8), and 10 mg/kg/d (LAMB10, *n* = 8), and untreated controls (UC, *n* = 14).

### Outcome variables

Quantitative clearance of *E. rostratum* from CNS tissues and declines of CSF (1→3)-β-d-glucan levels were used to assess in vivo antifungal activity.

*Quantitative cultures.* The representative sections of CNS tissues were obtained and processed, as previously described.^20^ Briefly, resected tissue sections were weighed, and homogenized with 2 or 5 mL of sterile 0.9% normal saline for 30 s.^22^ The aliquots (100 μL) of tissue homogenates and dilutions (10^−1^ and 10^−2^) were plated onto Sabouraud glucose agar plates and incubated at 37°C for 24 h. After 48 h, residual fungal colonies were counted and data were plotted as the mean log CFU/g ± standard error of the mean (SEM).

*(1→3)-β-D-glucan assay.* To determine CSF (1→3)-β-d-glucan levels, a sample from each rabbit was collected on the last day of experiment just after euthanasia. Levels of (1→3)-β-d-glucan were determined by assay according to the manufacturer's instructions (Fungitell^®^; Associates of Cape Cod, Inc., Falmouth, MA) as previously described.^20^ Briefly, a 96-well microtiter plate was filled with aliquots of 5 μL of CSF and pretreated for 10 min at 37°C with an alkaline reagent (20 μL; 0.125 M KOH/0.6 M KCl). An aliquot of 25 μL of the standards was then added to wells designated for the standard curve. An aliquot of 100 μL of mixture of Fungitell^®^ reagent (reconstituted with 2.8 mM glucan-free reagent-grade water plus 2.8 mL Pyrosol reconstitution buffer) was added to each sample. The plate was monitored at 405 nm minus 490 nm for 40 min at 37°C by using a Bio-Tek ELx808 automated microplate reader (Bio-Tek Instruments, Inc., Winooski, VT). The mean rate of optical density change was determined for each well, and the glucan concentration was determined by comparison to the standard curve. When absorbance was outside the range of the standard curve, the CSF samples were serially diluted in reagent-grade water and tested again. Values were plotted as mean (1→3)-β-d-glucan concentrations in picograms per milliliter in CSF for each study group.

### Statistical analysis

In vitro interaction for combinations was assessed by Bliss surface interaction analysis, as previously described.^16^ Comparisons between groups in vivo were performed by using the nonparametric analysis of Mann-Whitney *U*-test. A two-tailed *P* value of ≤ 0.05 was considered to be statistically significant. Since, there were no significant differences in outcome variables between *E. rostratum* isolates 12–2725 and 12–2809 used in in vivo experiments, data were pooled together. Values are expressed as means and standard error of the means (SEMs).

## Results

### In vitro combination studies

There was no synergistic or antagonistic interaction for combinations of isavuconazole plus micafungin and of isavuconazole plus DAMB against clinical isolates of *E. rostratum* as measured by MIC and FICI indices (Table 1)*.* Similar data demonstrating no synergistic or antagonistic interaction was observed when measured by Bliss surface interaction analysis. Subsequent studies of antifungal efficacy in vivo of *E. rostratum* meningoencephalitis experimental rabbit model were thus conducted with single agent monotherapy of isavuconazole or LAMB.

**Table 1.** Minimum inhibitory concentrations (MIC), minimum lethal concentrations (MLC), Bliss Surface Analysis Values, and fractional inhibitory concentration indifference (FICI) indices for combinations of isavuconazole (ISAV) plus micafungin (MFG), and ISAV plus amphotericin B deoxycholate (DAMB).

	(MIC; MLC) (μg/mL)			Combination (I-Independent)	
*E. rostratum* isolate	ISAV	MFG	DAMB	ISAV + MFG Bliss; FICI	ISAV + DAMB Bliss; FICI
12-2725	(0.5; >4.0)	(0.25; >8.0)	(0.125-0.25; 1.0)	(I; 0.49)	(I; 0.75)
12-2786	(4.0; 4.0)	(0.25; >8.0)	(0.03-0.06; 0.125)	(I; N/A)	(I; N/A)
12-2838	(1.0-2.0; >4.0)	(2.0-4.0; >8.0)	(0.06; >8.0)	(I; 1.0)	(I; 1.06)
12-2809	(4.0; >4.0)	(>8.0; >8.0)	(0.25; 2.0)	(I; 0.88)	(I; 1.03)
12-2840	(2.0; >4.0)	(0.06; >8.0)	(0.06; 2.0)	(I; N/A)	(I; N/A)

FIC indices: fractional inhibitory concentration; I-Independent interaction.

### Inoculum–response studies

**Figure 1.**
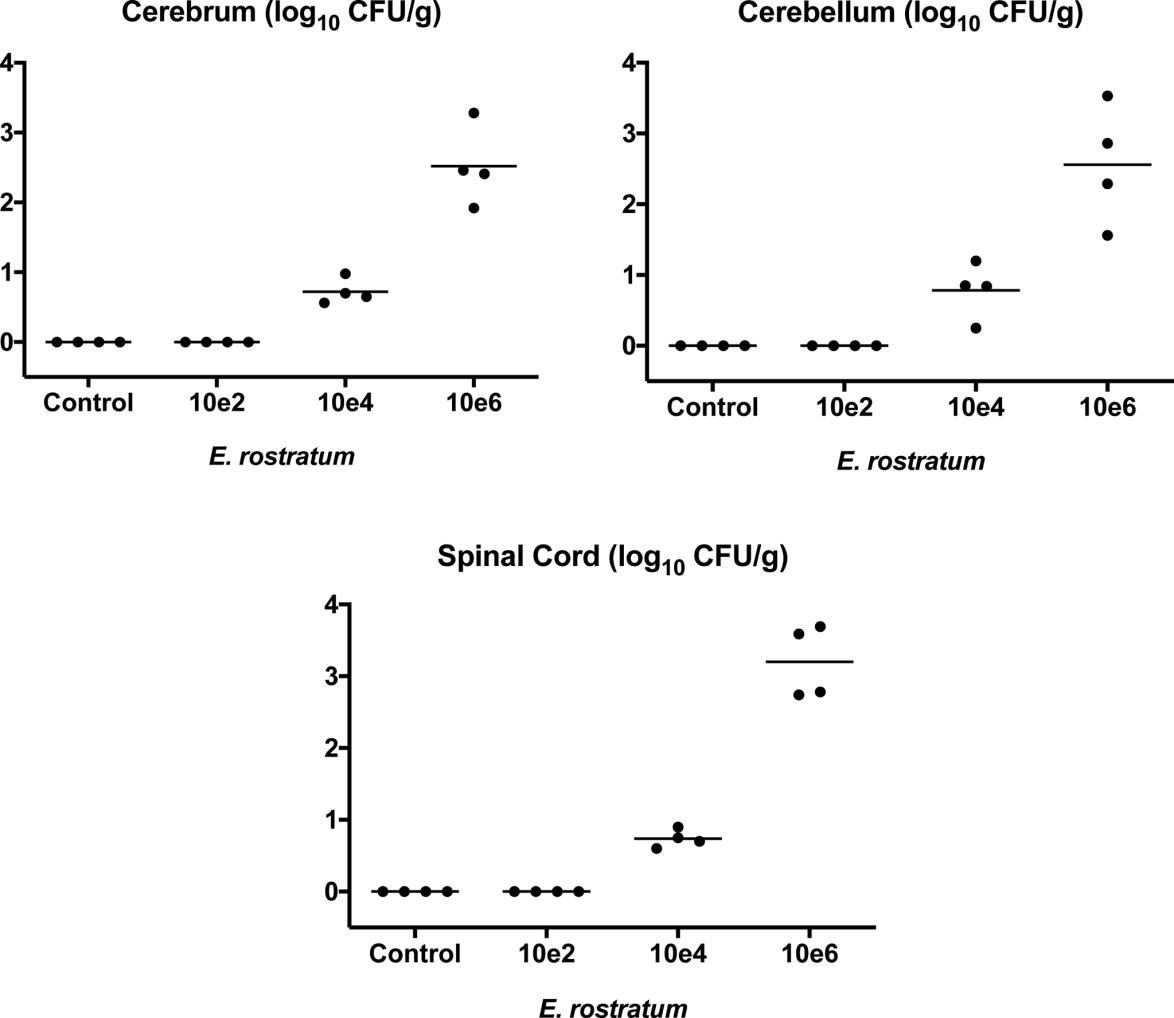
Scattergrams of inoculum–response, as measured by CFU/g in CNS tissues, including cerebrum, cerebellum, and spinal cord, versus CFU/inoculum of *Exserohilum rostratum* in immunocompromised rabbit model of meningoencephalitis. A bar through the column of each group represents the median log_10_ CFU/g for that group. There were four rabbits in each of three inoculum groups. Data were pooled from *E. rostratum* 12–2725 and *E. rostratum* 12–2809 results.

There was a direct inoculum-response relationship. A threshold intracisternal inoculum of ≥10^4^ conidia was required to establish experimental *E. rostratum* meningoencephalitis. A higher inoculum was associated with a significantly greater level of residual fungal burden in cerebral, cerebellar, and spinal cord tissues. Based on these results, an inoculum of 1.0 × 10^6^ conidia was selected for the efficacy studies in rabbits (Figure 1).

### In vivo efficacy studies

**Figure 2.**
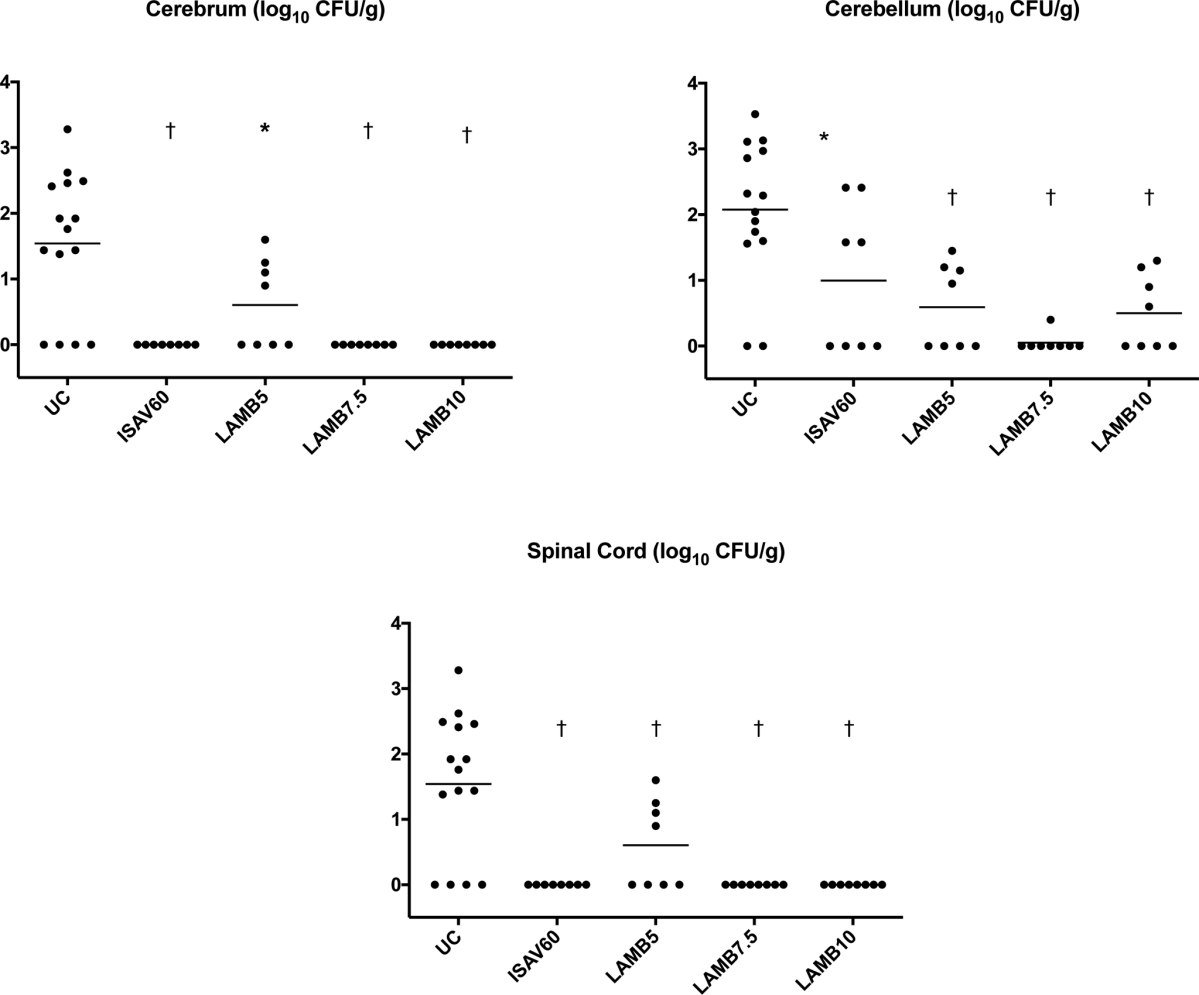
Scattergrams of response to antifungal therapy in persistently neutropenic rabbit model of *Exserohilum rostratum* meningoencephalitis. Antifungal efficacy was measured by mean tissue residual fungal burden (log CFU/g) in CNS tissues, including cerebrum, cerebellum, and spinal cord. Significant decrease in residual fungal burden in rabbits treated with isavuconazole at 60 mg/kg/d, liposomal amphotericin B at 5, 7.5, and 10 mg/kg/d were observed when compared to untreated controls (UC). There were eight rabbits (four rabbits per each isolate) in each of treatment groups, and 14 untreated controls. Data were pooled from *E. rostratum* 12–2725 and *E. rostratum* 12–2809 results. A bar through the column of each group represents the median log_10_ CFU/g for that group. ^*^P < 0.05; ^†^*P* < 0.01.

Rabbits treated with isavuconazole at 60 mg/kg/d, LAMB at 5 mg/kg/d, 7.5 mg/kg/d, and 10 mg/kg/d demonstrated a significant decrease of fungal burden in cerebral, cerebellar, and spinal cord tissues (*P* < 0.05), when compared to UC (Figure 2). The efficacy of isavuconazole at 60 mg/kg/d was comparable in efficacy to that of LAMB at 5, 7.5, and 10 mg/kg/d. Although two isolates were selected with low and high MICs for in vivo studies, there was no significant difference in therapeutic response between the isolates.

**Figure 3.**
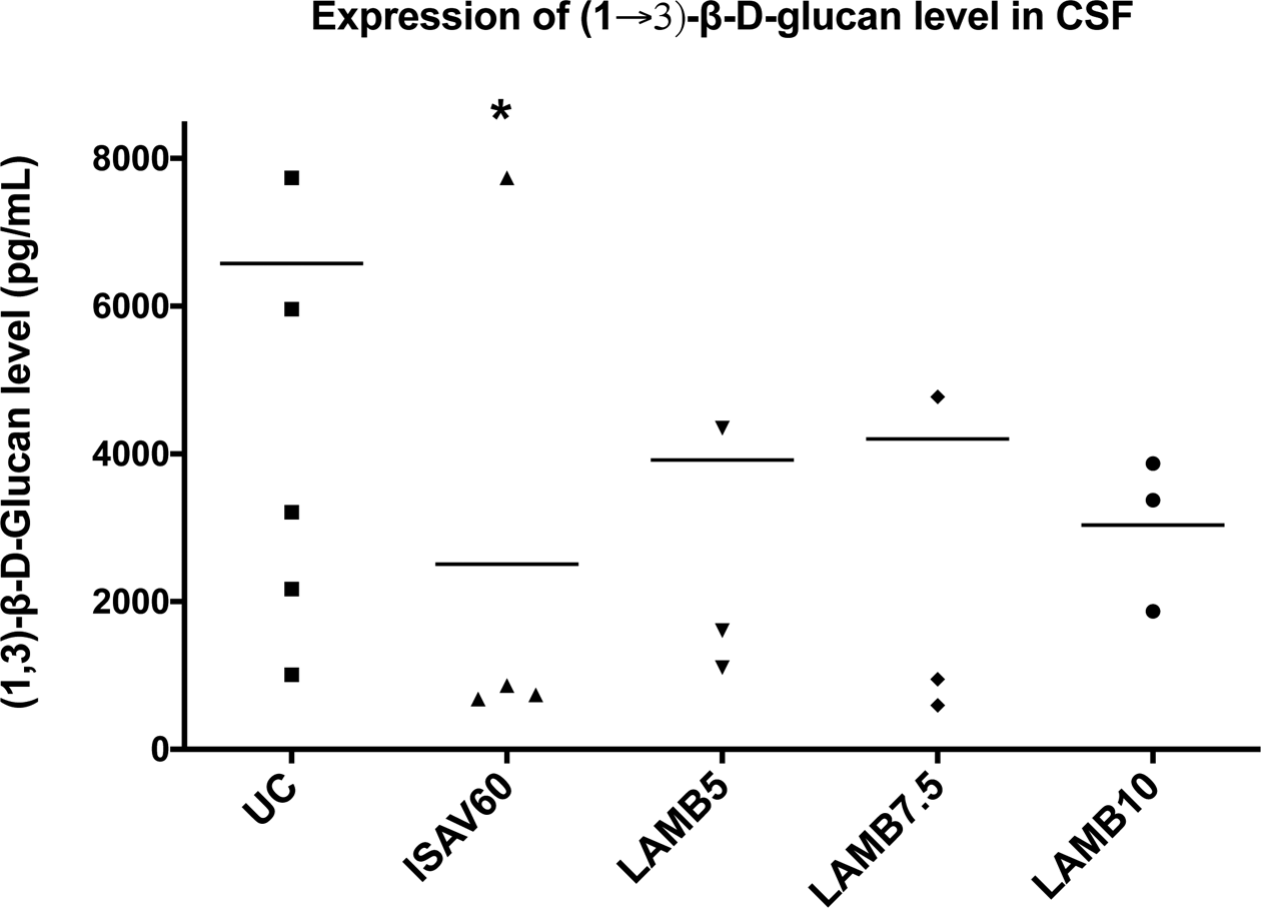
Scattergrams of detected CSF (1→3)-β-d-glucan levels in persistently neutropenic rabbit model of *Exserohilum rostratum* meningoencephalitis in untreated controls (UC, *n* = 5), and rabbits treated orally with isavuconazole at 60 mg/kg/d, and intravenously (*n* = 4) with LAMB at 5 mg/kg/d (*n* = 3), 7.5 mg/kg/d (*n* = 3), and 10 mg/kg/d (*n* = 3). Values are given as (1→3)-β-d-glucan concentrations picograms per milliliter. ^*^P < 0.05: significant decrease of CSF (1→3)-β-d-glucan levels in rabbits treated with isavuconazole at 60 mg/kg/d in comparison to that of UC. Data were pooled from results of *E. rostratum* isolates 12–2725 and 12–2809. A bar through the column of each group represents the median (1→3)-β-d-glucan level (pg/mL) for that group.

There were significant reductions in CSF (1→3)-β-d-glucan levels in rabbits treated with isavuconazole at 60 mg/kg/d, when compared to UC (*P* < 0.05). There also was a trend toward decreased levels of CSF (1→3)-β-d-glucan in rabbits treated with LAMB at 5, 7.5, and 10 mg/kg/d; however, these values did not reach statistical significance (Figure 3).

## Discussion

This study demonstrated that monotherapy with isavuconazole or LAMB significantly reduced residual fungal burden in cerebral, cerebellar, and spinal cord tissues in parallel with decreased CSF (1→3)-β-d-glucan levels in experimental *E. rostratum* CNS phaeohyphomycosis. Both isavuconazole and LAMB were comparable in efficacy in reduction of residual tissue burden in treatment of *E. rostratum* meningoencephalitis-related CNS phaeohyphomycosis. In vitro microdilution checkerboard studies demonstrated that the combination of isavuconazole plus micafungin or isavuconazole plus DAMB showed no synergistic or antagonistic interaction, thus warranting study of in vivo monotherapy. Results from this study provide a translational rationale for further study of isavuconazole or LAMB as monotherapy for treatment of CNS phaeohyphomycosis.

Isavuconazole possesses considerable advantage over voriconazole for the potential of long-term usage in treatment of CNS phaeohyphomycosis.^23^ A randomized trial of isavuconazole versus voriconazole demonstrated significantly fewer adverse events related to clinical toxicity, including hepatic injury, photopsia, visual hallucinations, and cutaneous hypersensitivity.^6^ Moreover, in most patients, isavuconazole has a predictable pharmacokinetic profile that minimizes the need for serial therapeutic drug monitoring.^24^ Finally, isavuconazole, which is metabolized primarily through CYP3A4, has fewer drug–drug interactions compared to that of voriconazole, which is metabolized through CYP3A4, CYP2C9, and CYP2C19.

LAMB also was effective as monotherapy at dosages ranging from 5 to 10 mg/kg/d. Responses were similar across the dosage range of 5 to 10 mg/kg/d. As the plasma exposures of LAMB achieved in the rabbit model are similar to those in humans, a dosage of 5 mg/kg/d can be considered in treatment of human CNS infection, thus avoiding higher dosages, which show dose-dependent-nephrotoxicity. Other studies against invasive aspergillosis have found that dosages >5mg/kg/d may not be more effective but may be more nephrotoxic.^25^^,^^26^ Moreover, isavuconazole would have an overall clinical advantage with minimal nephrotoxicity, as it demonstrated in the study herein that is comparable therapeutically to LAMB.

All isolates of *E. rostratum* that were investigated in this study were recovered from patients infected by contaminated corticosteroid injections related to the outbreak. A full background of each patient's clinical history for each respective isolate was not known. Among possible explanations of the inter-isolate variability of MICs for selected compounds are antifungal exposures before recovery of the organism, micro-evolution in the inanimate environment of the solution, or extant heteroresistance among the isolates.

The potential for combination therapy of isavuconazole plus micafungin was investigated predicated on the observations for the combination of micafungin plus isavuconazole against *A. fumigatus* in vitro and invasive pulmonary aspergillosis in vivo. The synergistic interaction between mould-active triazoles and echinocandins against *A. fumigatus* has been well documented in repeated studies in vitro and in vivo, particularly in the persistently neutropenic rabbit invasive pulmonary aspergillosis.^17^^,^^27^^,^^28^ These in vitro and in vivo findings were predictive of the outcome of the randomized controlled clinical trial of voriconazole plus anidulafungin versus voriconazole alone. This study demonstrated a trend toward improved fungal free survival in the primary endpoint, and in the post hoc test analysis showed significant improvement of survival in patients with probable invasive pulmonary aspergillosis with galactomannan-positive infection.^29^

The paucity of a synergistic interaction between isavuconazole and micafungin against *E. rostratum* underscores the importance for investigation of potential synergistic combinations against different non-*Aspergillus* moulds. While the synergistic combination of mould-active triazole and echinocandin are well documented for *Aspergillus fumigatus* and invasive pulmonary aspergillosis, differences in antifungal efficacy in phaeohyphomycetes may be related to the presence of melanin in cell walls, which may prevent a synergistic effect.

A direct inoculum response relationship was observed from 1.0 × 10^2^ to 1.0 × 10^6^ CFU in the model of *E. rostratum* meningoencephalitis with a threshold for infection at ≥1.0 × 10^4^ CFU. This observation may have important implications for understanding the infection dynamics of the multi-state outbreak of *E. rostratum* meningoencephalitis that infected more than 500 patients.^12^ During the outbreak, direct inoculation of contaminated solutions of methylprednisolone to approximately 14 000 patients occurred, yet most did not develop a clinically overt infection. Among the potential explanations for this observation may have been variable contamination of the vials with quantitatively higher levels of contamination accounting for those patients who developed clinical evidence of infection, where a threshold for infection was achieved. Other factors that would also have contributed may have been injected volume, the number of repeated injections, and host factors that may have increased the risk of disease.

Some patients with *E. rostratum* meningoencephalitis were reported to have paravertebral muscle infection. We therefore conducted a pilot investigation of paravertebral muscle infection within this study. These experiments demonstrated paravertebral muscle tissue infection in the model, further reflecting the similarity of this model to that of human infection.

Phaeohyphomycosis of the CNS is caused by a wide range of dematiaceous moulds. With more than 600 patients with infection of the brain and/or spinal cord caused by *E. rostratum*,^4^^,^^30^ this pathogen has caused the most recorded cases of CNS phaeohyphomycosis of all dematiaceous moulds. We therefore replicated the pattern of CNS infection caused by *E. rostratum* and investigated it for treatment of CNS phaeohyphomycosis. Other brown/black pigmented moulds may also be modeled; however, some pathogens would require a different route of inoculation; for example, *Cladophialophora bantiana*, which was the most common cause of CNS phaeohyphomycosis in the prospective series by Revankar et al,^4^ presents typically as solitary or multiple brain abscesses with minimal meningeal involvement.^31^^-^^33^ Thus, *Cladophialophora bantiana* CNS phaeohyphomycosis would likely be modeled as a pulmonary or intravenous portal of entry with hematogenous dissemination.^34^^,^^35^

In summary, isavuconazole at 60 mg/kg/d, LAMB at 5.0 mg/kg/d, LAMB 7.5 mg/kg/d, and LAMB 10 mg/kg/d as monotherapies were found to significantly decrease the residual fungal burden of *E. rostratum* by as much as 100-fold in cerebral, cerebellar, and spinal cord tissues. We conclude that isavuconazole is a promising agent with in vivo activity comparable to that of LAMB in treatment of *E. rostratum* meningoencephalitis and may be effective in other forms of CNS phaeohyphomycosis.
